# Refractory Headache: One Term does Not cover All – A Statement of the European Headache Federation

**DOI:** 10.1186/1129-2377-15-50

**Published:** 2014-08-28

**Authors:** Christian Lampl, Rigmor Jensen, Paolo Martelletti, Dimos-Dimitrios Mitsikostas

**Affiliations:** 1 Headache Medical Centre, Seilerstaette Linz, Hospital Barmherzige Schwestern, Linz, Austria; 2 Danish Headache Centre, Glostrup Hospital, University of Copenhagen, Copenhagen, Denmark; 3 Department of Clinical and Molecular Medicine, Sapienza University of Rome, Rome, Italy; 4 Department of Neurology, Athens Naval Hospital, Athens, Greece

## 

In the past years a unifying definition of refractory headache (rH) has been extensively discussed [1,2] but, to date, has not been agreed upon. It is widely agreed, that refractoriness, for whatever category and disease, implies a high burden with tremendous impact in health related quality of life (HRQoL) [3]. Despite that fact, an overall accepted definition of rH would be more than important for managing and triaging patients to an appropriate level of care and for determining eligibility for epidemiological and clinical studies.

So far, there are different and non-conclusive categories that try to describe refractoriness. In the understanding of refractoriness particular in headache patients several important issues have to be addressed: First, it is of importance to emphasize the difficulty that refractoriness in headache may just represent more or less treatable version(s) of many different disorders, rather than a unique disease or group of disorders. Second, the same patient might be identified as refractory at one time, but treatment responsive at another. Therefore it may be of crucial importance to evaluate acute and prophylactic treatment response, baseline headache severity, partial response versus an all-or-none response, and the possibility of any variability in the treatment response over time for each headache disorder and patient from the very first on. Some evidence supports the hypothesis that baseline headache attack intensity has an impact on determination of treatment response [4]. It may also be hypothesized that patients with high baseline headache frequency were more likely to be drug resistant, meaning that it is harder to eradicate many headache attacks than a few. Without recording baseline frequency and severity rate, it is almost impossible to know whether there has been partial or no response to treatment. But that’s the crucial point: clinicians have the need for medical treatment mostly before severity of headache and frequency of disease can be determined. When headache attacks are not completely controlled, the conclusion may be that the administered drug is not effective and that the patient therefore is “resistant”. Third, as in other conditions [5] the definition of responder or non-responder enormously differs among both clinicians and investigators.

All these considerations lead to variability in clinical and epidemiological research results, both being of importance to increase our knowledge in the understanding of rH. What are the critical issues so far: (i) there is no standardized definition of rH; (ii) at the time of first diagnosis headache patients do not necessarily become refractory immediately, nor do they mandatorily remain refractory throughout the course of their disease; (iii) due to the necessity that most patients should be treated rapidly after diagnosis response to medication often is assessed without a pretreatment baseline and it remains unclear whether or not so-called refractory patients have had a substantial response to treatment; (iv) headache pain and associated symptoms are frequently intermittent, making this disease different from others that have been examined for treatment resistance; (v) the natural history is not known.

For all these purposes the Board of the European Headache Federation (EHF) felt the need to develop new consensus criteria that define refractory chronic migraine (rCM) and refractory chronic cluster headache (rCCH). These new definitions of rCM and rCCH, which were agreed upon within the EHF, allows us to separate patients into categories of refractory and non-refractory, being important for clinicians, clinical and epidemiological trials.

The EHF is aware of that still many challenges are on the road in identifying which patients are really treatment resistant and to what degree. It is a misconception that a patient necessarily will fall into one of the two categories and stay there. From a clinical perspective a large number of patients may fit each category for periods of time. But patients may also move in both directions, from refractory to responsive and the opposite. E.g. after neuromodulation, many patients will become headache free but have to continue with prophylactic medication to prevent headache recurrence. These patients have shifted from being treatment resistant to being treatment sensitive.

To define treatment response - EHF claims the need to go into patient categorization - one term does not fit all. We badly need the same definitions of rH, better information about pretreatment headache rate and severity, more precise information about prior acute and prophylactic treatment response and scientific data regarding the natural history of drug response.
